# Supplementary material for: Modeling glioblastoma heterogeneity as a dynamic network of cell states
Source: Mol Syst Biol. 2021 Sep 16;17(9):e10105. doi: 10.15252/msb.202010105 (PMC8444284; doi:10.15252/msb.202010105)
Supplement: Supplementary file 5 — Source Data for Figure 3 [file MSB-17-e10105-s001.zip › Figure3A_sourcedata/GSEA_3065/hallmarks_state6.GseaPreranked.1623416308702/HALLMARK_KRAS_SIGNALING_DN.html]

Details for gene set HALLMARK\_KRAS\_SIGNALING\_DN[GSEA]

|  || Dataset | state6 |
| Phenotype | NoPhenotypeAvailable |
| Upregulated in class | na\_pos |
| GeneSet | HALLMARK\_KRAS\_SIGNALING\_DN |
| Enrichment Score (ES) | 0.2847776 |
| Normalized Enrichment Score (NES) | 0.9719439 |
| Nominal p-value | 0.51972157 |
| FDR q-value | 0.5855652 |
| FWER p-Value | 1.0 |
Table: GSEA Results Summary

  

Fig 1: Enrichment plot: HALLMARK\_KRAS\_SIGNALING\_DN      
 Profile of the Running ES Score & Positions of GeneSet Members on the Rank Ordered List

  

| PROBE | GENE SYMBOL | GENE\_TITLE | RANK IN GENE LIST | RANK METRIC SCORE | RUNNING ES | CORE ENRICHMENT || 1 | BTG2 |  |  | 63 | 0.234 | 0.1161 | Yes |
| 2 | PDK2 |  |  | 426 | 0.131 | 0.1438 | Yes |
| 3 | TCF7L1 |  |  | 427 | 0.131 | 0.2127 | Yes |
| 4 | SNN |  |  | 473 | 0.126 | 0.2738 | Yes |
| 5 | SLC25A23 |  |  | 823 | 0.097 | 0.2848 | Yes |
| 6 | GTF3C5 |  |  | 1824 | 0.053 | 0.1984 | No |
| 7 | SKIL |  |  | 1921 | 0.050 | 0.2138 | No |
| 8 | NUDT11 |  |  | 1971 | 0.049 | 0.2337 | No |
| 9 | CCDC106 |  |  | 2556 | 0.032 | 0.1838 | No |
| 10 | KCNQ2 |  |  | 2653 | 0.030 | 0.1886 | No |
| 11 | CELSR2 |  |  | 2741 | 0.028 | 0.1934 | No |
| 12 | CAMK1D |  |  | 3613 | 0.009 | 0.0987 | No |
| 13 | COPZ2 |  |  | 3660 | 0.008 | 0.0975 | No |
| 14 | EFHD1 |  |  | 3739 | 0.006 | 0.0917 | No |
| 15 | CDKAL1 |  |  | 3904 | 0.002 | 0.0742 | No |
| 16 | GAMT |  |  | 4285 | -0.005 | 0.0333 | No |
| 17 | SLC38A3 |  |  | 4428 | -0.007 | 0.0210 | No |
| 18 | DCC |  |  | 4432 | -0.007 | 0.0246 | No |
| 19 | YPEL1 |  |  | 4712 | -0.013 | -0.0005 | No |
| 20 | CHST2 |  |  | 4821 | -0.015 | -0.0051 | No |
| 21 | MFSD6 |  |  | 4867 | -0.016 | -0.0019 | No |
| 22 | SPHK2 |  |  | 5447 | -0.027 | -0.0536 | No |
| 23 | PTPRJ |  |  | 5664 | -0.032 | -0.0615 | No |
| 24 | VPS50 |  |  | 5929 | -0.037 | -0.0724 | No |
| 25 | KLHDC8A |  |  | 6231 | -0.043 | -0.0840 | No |
| 26 | BARD1 |  |  | 6284 | -0.044 | -0.0667 | No |
| 27 | TGFB2 |  |  | 7048 | -0.066 | -0.1189 | No |
| 28 | SHOX2 |  |  | 7163 | -0.070 | -0.0952 | No |
| 29 | KMT2D |  |  | 7227 | -0.072 | -0.0646 | No |
| 30 | LFNG |  |  | 7578 | -0.085 | -0.0599 | No |
| 31 | FGFR3 |  |  | 8163 | -0.118 | -0.0643 | No |
| 32 | IGFBP2 |  |  | 8719 | -0.260 | 0.0088 | No |
Table: GSEA details [plain text format]

  

Fig 2: HALLMARK\_KRAS\_SIGNALING\_DN: Random ES distribution      
 Gene set null distribution of ES for **HALLMARK\_KRAS\_SIGNALING\_DN**
